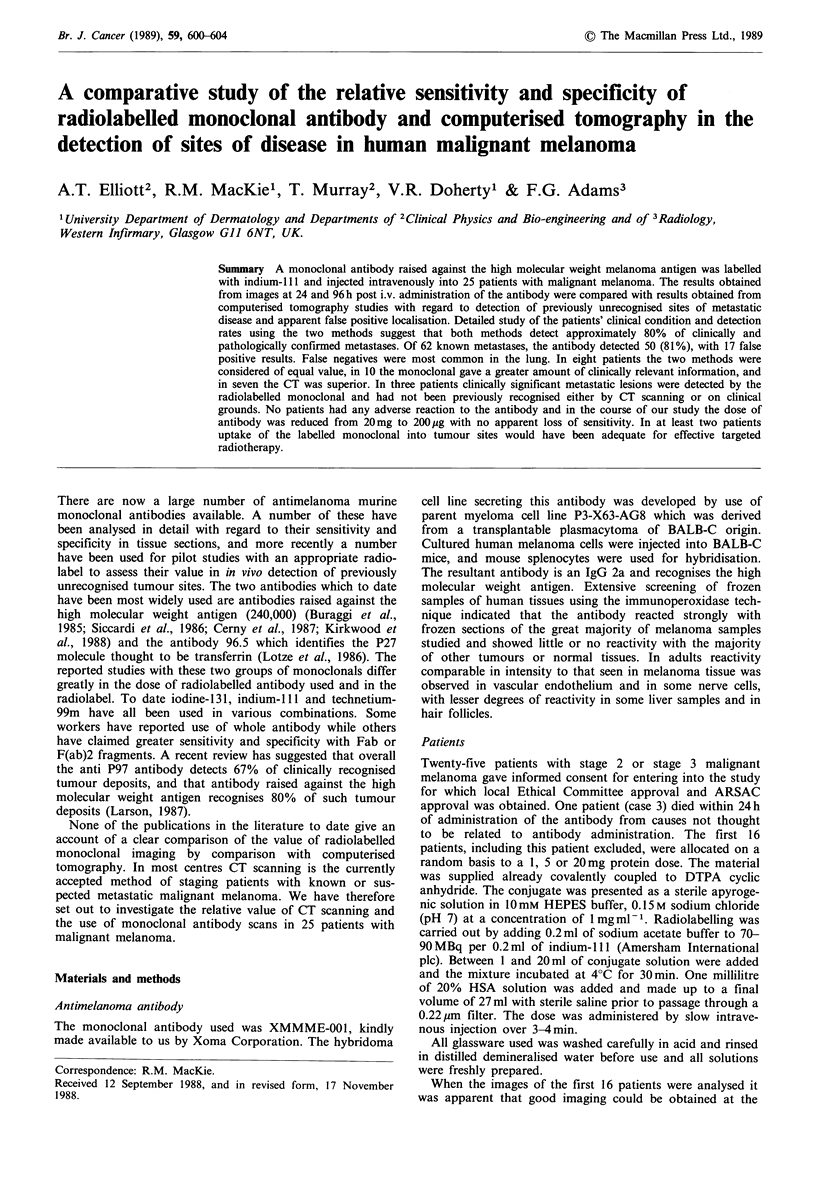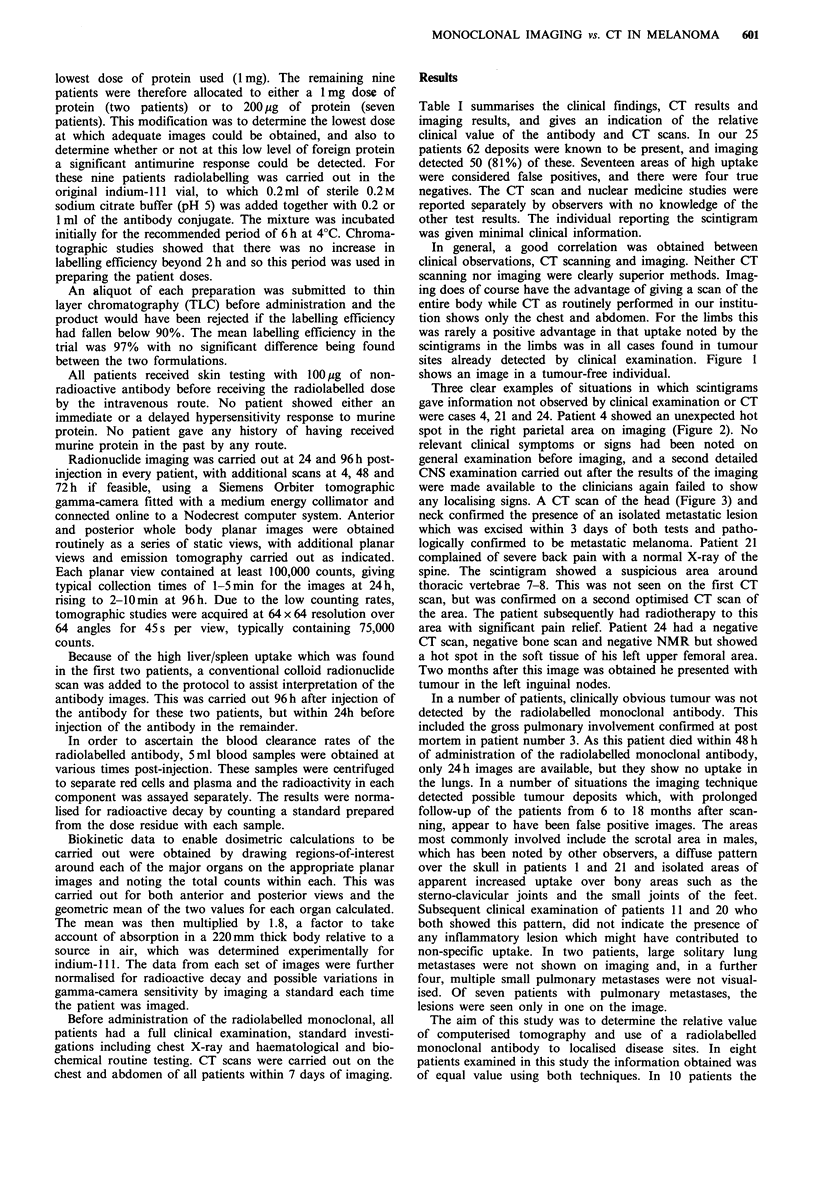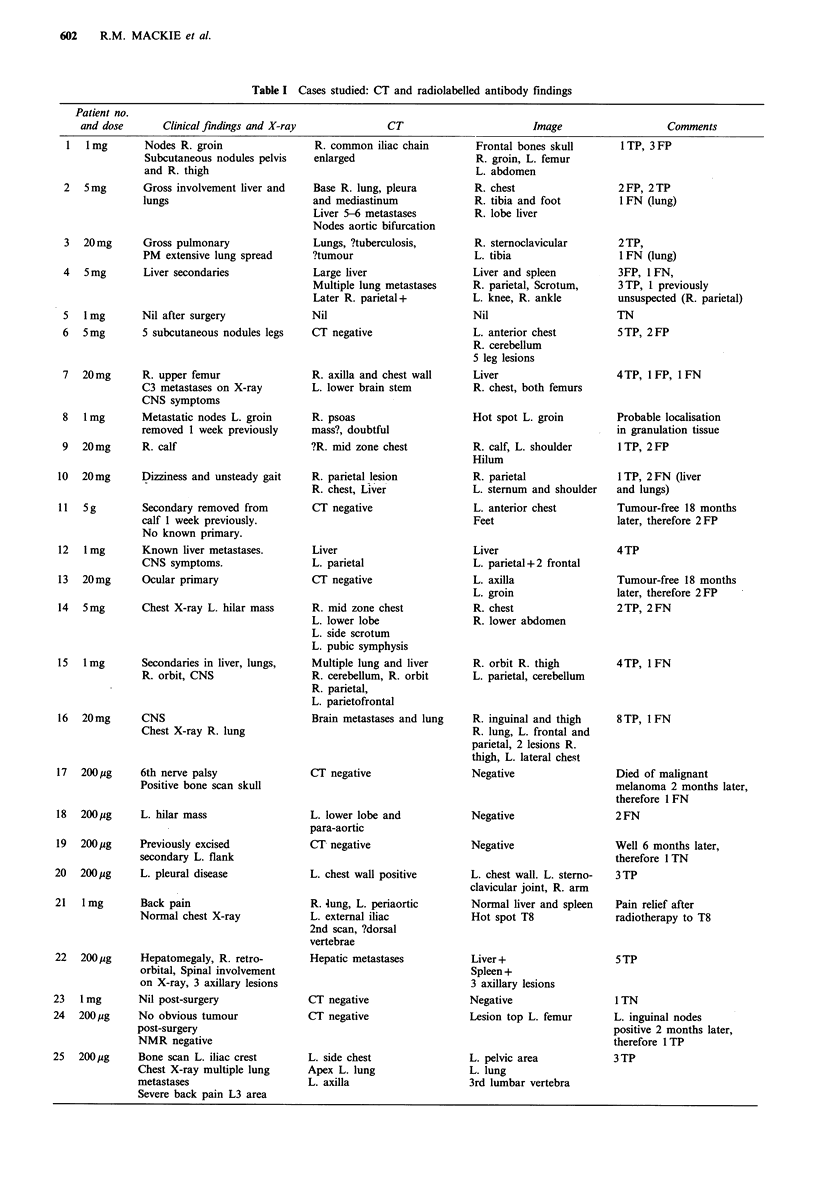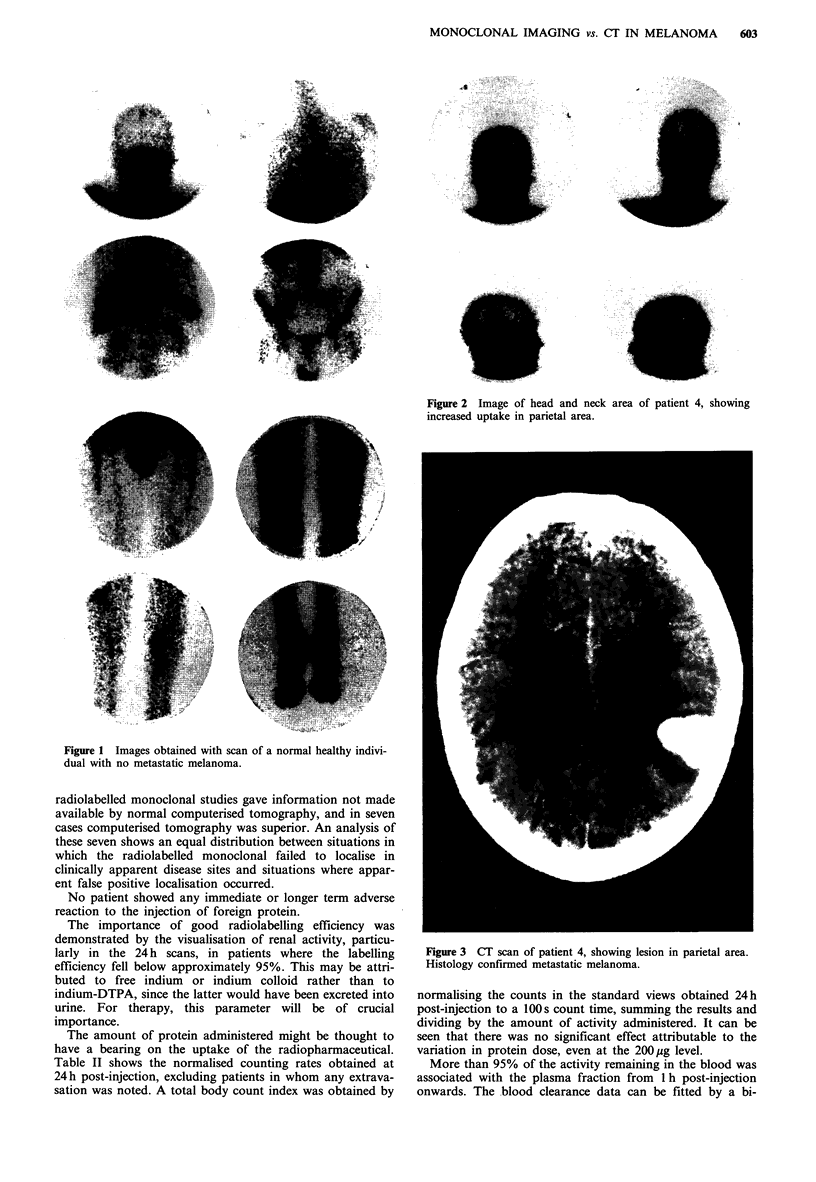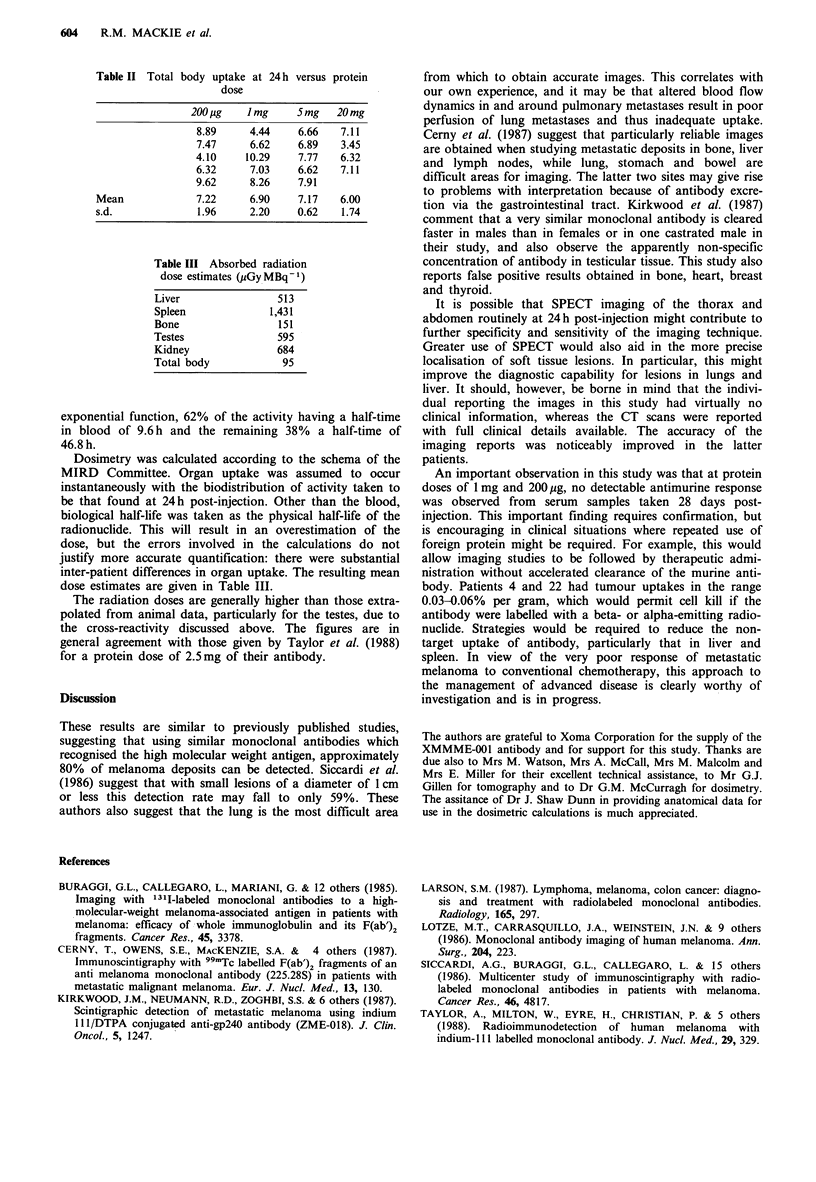# A comparative study of the relative sensitivity and specificity of radiolabelled monoclonal antibody and computerised tomography in the detection of sites of disease in human malignant melanoma.

**DOI:** 10.1038/bjc.1989.121

**Published:** 1989-04

**Authors:** A. T. Elliott, R. M. MacKie, T. Murray, V. R. Doherty, F. G. Adams

**Affiliations:** University Department of Dermatology, Western Infirmary, Glasgow, UK.

## Abstract

**Images:**


Br. J. Cancer (1989), 59, 600–604 © The Macmillan Press Ltd., 1989

### A comparative study of the relative sensitivity and specificity of radiolabelled monoclonal antibody and computerised tomography in the detection of sites of disease in human malignant melanoma

A.T. Elliott[2], R.M. MacKie[1], T. Murray[2], V.R. Doherty[1] & F.G. Adams[3]

[1]*University Department of Dermatology and Departments of* [2]*Clinical Physics and Bio-engineering and of* [3]*Radiology, Western Infirmary, Glasgow G11 6NT, UK.*

**Summary** A monoclonal antibody raised against the high molecular weight melanoma antigen was labelled with indium-111 and injected intravenously into 25 patients with malignant melanoma. The results obtained from images at 24 and 96 h post i.v. administration of the antibody were compared with results obtained from computerised tomography studies with regard to detection of previously unrecognised sites of metastatic disease and apparent false positive localisation. Detailed study of the patients' clinical condition and detection rates using the two methods suggest that both methods detect approximately 80% of clinically and pathologically confirmed metastases. Of 62 known metastases, the antibody detected 50 (81%), with 17 false positive results. False negatives were most common in the lung. In eight patients the two methods were considered of equal value, in 10 the monoclonal gave a greater amount of clinically relevant information, and in seven the CT was superior. In three patients clinically significant metastatic lesions were detected by the radiolabelled monoclonal and had not been previously recognised either by CT scanning or on clinical grounds. No patients had any adverse reaction to the antibody and in the course of our study the dose of antibody was reduced from 20 mg to 200 $\mu$g with no apparent loss of sensitivity. In at least two patients uptake of the labelled monoclonal into tumour sites would have been adequate for effective targeted radiotherapy.

There are now a large number of antimelanoma murine monoclonal antibodies available. A number of these have been analysed in detail with regard to their sensitivity and specificity in tissue sections, and more recently a number have been used for pilot studies with an appropriate radiolabel to assess their value in *in vivo* detection of previously unrecognised tumour sites. The two antibodies which to date have been most widely used are antibodies raised against the high molecular weight antigen (240,000) (Buraggi *et al.*, 1985; Siccardi *et al.*, 1986; Cerny *et al.*, 1987; Kirkwood *et al.*, 1988) and the antibody 96.5 which identifies the P27 molecule thought to be transferrin (Lotze *et al.*, 1986). The reported studies with these two groups of monoclonals differ greatly in the dose of radiolabelled antibody used and in the radiolabel. To date iodine-131, indium-111 and technetium-99m have all been used in various combinations. Some workers have reported use of whole antibody while others have claimed greater sensitivity and specificity with Fab or F(ab)2 fragments. A recent review has suggested that overall the anti P97 antibody detects 67% of clinically recognised tumour deposits, and that antibody raised against the high molecular weight antigen recognises 80% of such tumour deposits (Larson, 1987).

None of the publications in the literature to date give an account of a clear comparison of the value of radiolabelled monoclonal imaging by comparison with computerised tomography. In most centres CT scanning is the currently accepted method of staging patients with known or suspected metastatic malignant melanoma. We have therefore set out to investigate the relative value of CT scanning and the use of monoclonal antibody scans in 25 patients with malignant melanoma.

### Materials and methods

### *Antimelanoma antibody*

The monoclonal antibody used was XMMME-001, kindly made available to us by Xoma Corporation. The hybridoma cell line secreting this antibody was developed by use of parent myeloma cell line P3-X63-AG8 which was derived from a transplantable plasmacytoma of BALB-C origin. Cultured human melanoma cells were injected into BALB-C mice, and mouse splenocytes were used for hybridisation. The resultant antibody is an IgG 2a and recognises the high molecular weight antigen. Extensive screening of frozen samples of human tissues using the immunoperoxidase technique indicated that the antibody reacted strongly with frozen sections of the great majority of melanoma samples studied and showed little or no reactivity with the majority of other tumours or normal tissues. In adults reactivity comparable in intensity to that seen in melanoma tissue was observed in vascular endothelium and in some nerve cells, with lesser degrees of reactivity in some liver samples and in hair follicles.

### *Patients*

Twenty-five patients with stage 2 or stage 3 malignant melanoma gave informed consent for entering into the study for which local Ethical Committee approval and ARSAC approval was obtained. One patient (case 3) died within 24 h of administration of the antibody from causes not thought to be related to antibody administration. The first 16 patients, including this patient excluded, were allocated on a random basis to a 1, 5 or 20 mg protein dose. The material was supplied already covalently coupled to DTPA cyclic anhydride. The conjugate was presented as a sterile apyrogenic solution in 10 mM HEPES buffer, 0.15 M sodium chloride (pH 7) at a concentration of 1 mg ml$^{-1}$. Radiolabelling was carried out by adding 0.2 ml of sodium acetate buffer to 70–90 MBq per 0.2 ml of indium-111 (Amersham International plc). Between 1 and 20 ml of conjugate solution were added and the mixture incubated at 4°C for 30 min. One millilitre of 20% HSA solution was added and made up to a final volume of 27 ml with sterile saline prior to passage through a 0.22 $\mu$m filter. The dose was administered by slow intravenous injection over 3–4 min.

All glassware used was washed carefully in acid and rinsed in distilled demineralised water before use and all solutions were freshly prepared.

When the images of the first 16 patients were analysed it was apparent that good imaging could be obtained at the

Correspondence: R.M. MacKie.
Received 12 September 1988, and in revised form, 17 November 1988.

lowest dose of protein used (1 mg). The remaining nine patients were therefore allocated to either a 1 mg dose of protein (two patients) or to 200 $\mu$g of protein (seven patients). This modification was to determine the lowest dose at which adequate images could be obtained, and also to determine whether or not at this low level of foreign protein a significant antimurine response could be detected. For these nine patients radiolabelling was carried out in the original indium-111 vial, to which 0.2 ml of sterile 0.2 M sodium citrate buffer (pH 5) was added together with 0.2 or 1 ml of the antibody conjugate. The mixture was incubated initially for the recommended period of 6 h at 4°C. Chromatographic studies showed that there was no increase in labelling efficiency beyond 2 h and so this period was used in preparing the patient doses.

An aliquot of each preparation was submitted to thin layer chromatography (TLC) before administration and the product would have been rejected if the labelling efficiency had fallen below 90%. The mean labelling efficiency in the trial was 97% with no significant difference being found between the two formulations.

All patients received skin testing with 100 $\mu$g of non-radioactive antibody before receiving the radiolabelled dose by the intravenous route. No patient showed either an immediate or a delayed hypersensitivity response to murine protein. No patient gave any history of having received murine protein in the past by any route.

Radionuclide imaging was carried out at 24 and 96 h post-injection in every patient, with additional scans at 4, 48 and 72 h if feasible, using a Siemens Orbiter tomographic gamma-camera fitted with a medium energy collimator and connected online to a Nodecrest computer system. Anterior and posterior whole body planar images were obtained routinely as a series of static views, with additional planar views and emission tomography carried out as indicated. Each planar view contained at least 100,000 counts, giving typical collection times of 1–5 min for the images at 24 h, rising to 2–10 min at 96 h. Due to the low counting rates, tomographic studies were acquired at 64 x 64 resolution over 64 angles for 45 s per view, typically containing 75,000 counts.

Because of the high liver/spleen uptake which was found in the first two patients, a conventional colloid radionuclide scan was added to the protocol to assist interpretation of the antibody images. This was carried out 96 h after injection of the antibody for these two patients, but within 24h before injection of the antibody in the remainder.

In order to ascertain the blood clearance rates of the radiolabelled antibody, 5 ml blood samples were obtained at various times post-injection. These samples were centrifuged to separate red cells and plasma and the radioactivity in each component was assayed separately. The results were normalised for radioactive decay by counting a standard prepared from the dose residue with each sample.

Biokinetic data to enable dosimetric calculations to be carried out were obtained by drawing regions-of-interest around each of the major organs on the appropriate planar images and noting the total counts within each. This was carried out for both anterior and posterior views and the geometric mean of the two values for each organ calculated. The mean was then multiplied by 1.8, a factor to take account of absorption in a 220 mm thick body relative to a source in air, which was determined experimentally for indium-111. The data from each set of images were further normalised for radioactive decay and possible variations in gamma-camera sensitivity by imaging a standard each time the patient was imaged.

Before administration of the radiolabelled monoclonal, all patients had a full clinical examination, standard investigations including chest X-ray and haematological and biochemical routine testing. CT scans were carried out on the chest and abdomen of all patients within 7 days of imaging.

### Results

Table I summarises the clinical findings, CT results and imaging results, and gives an indication of the relative clinical value of the antibody and CT scans. In our 25 patients 62 deposits were known to be present, and imaging detected 50 (81%) of these. Seventeen areas of high uptake were considered false positives, and there were four true negatives. The CT scan and nuclear medicine studies were reported separately by observers with no knowledge of the other test results. The individual reporting the scintigram was given minimal clinical information.

In general, a good correlation was obtained between clinical observations, CT scanning and imaging. Neither CT scanning nor imaging were clearly superior methods. Imaging does of course have the advantage of giving a scan of the entire body while CT as routinely performed in our institution shows only the chest and abdomen. For the limbs this was rarely a positive advantage in that uptake noted by the scintigrams in the limbs was in all cases found in tumour sites already detected by clinical examination. Figure 1 shows an image in a tumour-free individual.

Three clear examples of situations in which scintigrams gave information not observed by clinical examination or CT were cases 4, 21 and 24. Patient 4 showed an unexpected hot spot in the right parietal area on imaging (Figure 2). No relevant clinical symptoms or signs had been noted on general examination before imaging, and a second detailed CNS examination carried out after the results of the imaging were made available to the clinicians again failed to show any localising signs. A CT scan of the head (Figure 3) and neck confirmed the presence of an isolated metastatic lesion which was excised within 3 days of both tests and pathologically confirmed to be metastatic melanoma. Patient 21 complained of severe back pain with a normal X-ray of the spine. The scintigram showed a suspicious area around thoracic vertebrae 7–8. This was not seen on the first CT scan, but was confirmed on a second optimised CT scan of the area. The patient subsequently had radiotherapy to this area with significant pain relief. Patient 24 had a negative CT scan, negative bone scan and negative NMR but showed a hot spot in the soft tissue of his left upper femoral area. Two months after this image was obtained he presented with tumour in the left inguinal nodes.

In a number of patients, clinically obvious tumour was not detected by the radiolabelled monoclonal antibody. This included the gross pulmonary involvement confirmed at post mortem in patient number 3. As this patient died within 48 h of administration of the radiolabelled monoclonal antibody, only 24 h images are available, but they show no uptake in the lungs. In a number of situations the imaging technique detected possible tumour deposits which, with prolonged follow-up of the patients from 6 to 18 months after scanning, appear to have been false positive images. The areas most commonly involved include the scrotal area in males, which has been noted by other observers, a diffuse pattern over the skull in patients 1 and 21 and isolated areas of apparent increased uptake over bony areas such as the sterno-clavicular joints and the small joints of the feet. Subsequent clinical examination of patients 11 and 20 who both showed this pattern, did not indicate the presence of any inflammatory lesion which might have contributed to non-specific uptake. In two patients, large solitary lung metastases were not shown on imaging and, in a further four, multiple small pulmonary metastases were not visualised. Of seven patients with pulmonary metastases, the lesions were seen only in one on the image.

The aim of this study was to determine the relative value of computerised tomography and use of a radiolabelled monoclonal antibody to localised disease sites. In eight patients examined in this study the information obtained was of equal value using both techniques. In 10 patients the

**Table I** Cases studied: CT and radiolabelled antibody findings

| *Patient no. and dose* | | *Clinical findings and X-ray* | *CT* | *Image* | *Comments* |
|---|---|---|---|---|---|
| 1 | 1 mg | Nodes R. groin<br>Subcutaneous nodules pelvis and R. thigh | R. common iliac chain enlarged | Frontal bones skull<br>R. groin, L. femur<br>L. abdomen | 1 TP, 3 FP |
| 2 | 5 mg | Gross involvement liver and lungs | Base R. lung, pleura and mediastinum<br>Liver 5–6 metastases<br>Nodes aortic bifurcation | R. chest<br>R. tibia and foot<br>R. lobe liver | 2 FP, 2 TP<br>1 FN (lung) |
| 3 | 20 mg | Gross pulmonary<br>PM extensive lung spread | Lungs, ?tuberculosis, ?tumour | R. sternoclavicular<br>L. tibia | 2 TP,<br>1 FN (lung) |
| 4 | 5 mg | Liver secondaries | Large liver<br>Multiple lung metastases<br>Later R. parietal+ | Liver and spleen<br>R. parietal, Scrotum,<br>L. knee, R. ankle | 3FP, 1 FN,<br>3 TP, 1 previously unsuspected (R. parietal) |
| 5 | 1 mg | Nil after surgery | Nil | Nil | TN |
| 6 | 5 mg | 5 subcutaneous nodules legs | CT negative | L. anterior chest<br>R. cerebellum<br>5 leg lesions | 5 TP, 2 FP |
| 7 | 20 mg | R. upper femur<br>C3 metastases on X-ray<br>CNS symptoms | R. axilla and chest wall<br>L. lower brain stem | Liver<br>R. chest, both femurs | 4 TP, 1 FP, 1 FN |
| 8 | 1 mg | Metastatic nodes L. groin removed 1 week previously | R. psoas mass?, doubtful | Hot spot L. groin | Probable localisation in granulation tissue |
| 9 | 20 mg | R. calf | ?R. mid zone chest | R. calf, L. shoulder<br>Hilum | 1 TP, 2 FP |
| 10 | 20 mg | Dizziness and unsteady gait | R. parietal lesion<br>R. chest, Liver | R. parietal<br>L. sternum and shoulder | 1 TP, 2 FN (liver and lungs) |
| 11 | 5 g | Secondary removed from calf 1 week previously.<br>No known primary. | CT negative | L. anterior chest<br>Feet | Tumour-free 18 months later, therefore 2 FP |
| 12 | 1 mg | Known liver metastases.<br>CNS symptoms. | Liver<br>L. parietal | Liver<br>L. parietal + 2 frontal | 4 TP |
| 13 | 20 mg | Ocular primary | CT negative | L. axilla<br>L. groin | Tumour-free 18 months later, therefore 2 FP |
| 14 | 5 mg | Chest X-ray L. hilar mass | R. mid zone chest<br>L. lower lobe<br>L. side scrotum<br>L. pubic symphysis | R. chest<br>R. lower abdomen | 2 TP, 2 FN |
| 15 | 1 mg | Secondaries in liver, lungs, R. orbit, CNS | Multiple lung and liver<br>R. cerebellum, R. orbit<br>R. parietal,<br>L. parietofrontal | R. orbit R. thigh<br>L. parietal, cerebellum | 4 TP, 1 FN |
| 16 | 20 mg | CNS<br>Chest X-ray R. lung | Brain metastases and lung | R. inguinal and thigh<br>R. lung, L. frontal and parietal, 2 lesions R. thigh, L. lateral chest | 8 TP, 1 FN |
| 17 | 200 μg | 6th nerve palsy<br>Positive bone scan skull | CT negative | Negative | Died of malignant melanoma 2 months later, therefore 1 FN |
| 18 | 200 μg | L. hilar mass | L. lower lobe and para-aortic | Negative | 2 FN |
| 19 | 200 μg | Previously excised secondary L. flank | CT negative | Negative | Well 6 months later, therefore 1 TN |
| 20 | 200 μg | L. pleural disease | L. chest wall positive | L. chest wall. L. sterno-clavicular joint, R. arm | 3 TP |
| 21 | 1 mg | Back pain<br>Normal chest X-ray | R. lung, L. periaortic<br>L. external iliac<br>2nd scan, ?dorsal vertebrae | Normal liver and spleen<br>Hot spot T8 | Pain relief after radiotherapy to T8 |
| 22 | 200 μg | Hepatomegaly, R. retro-orbital, Spinal involvement on X-ray, 3 axillary lesions | Hepatic metastases | Liver+<br>Spleen+<br>3 axillary lesions | 5 TP |
| 23 | 1 mg | Nil post-surgery | CT negative | Negative | 1 TN |
| 24 | 200 μg | No obvious tumour post-surgery<br>NMR negative | CT negative | Lesion top L. femur | L. inguinal nodes positive 2 months later, therefore 1 TP |
| 25 | 200 μg | Bone scan L. iliac crest<br>Chest X-ray multiple lung metastases<br>Severe back pain L3 area | L. side chest<br>Apex L. lung<br>L. axilla | L. pelvic area<br>L. lung<br>3rd lumbar vertebra | 3 TP |

**Figure 1** Images obtained with scan of a normal healthy individual with no metastatic melanoma.

**Figure 2** Image of head and neck area of patient 4, showing increased uptake in parietal area.

**Figure 3** CT scan of patient 4, showing lesion in parietal area. Histology confirmed metastatic melanoma.

radiolabelled monoclonal studies gave information not made available by normal computerised tomography, and in seven cases computerised tomography was superior. An analysis of these seven shows an equal distribution between situations in which the radiolabelled monoclonal failed to localise in clinically apparent disease sites and situations where apparent false positive localisation occurred.

No patient showed any immediate or longer term adverse reaction to the injection of foreign protein.

The importance of good radiolabelling efficiency was demonstrated by the visualisation of renal activity, particularly in the 24 h scans, in patients where the labelling efficiency fell below approximately 95%. This may be attributed to free indium or indium colloid rather than to indium-DTPA, since the latter would have been excreted into urine. For therapy, this parameter will be of crucial importance.

The amount of protein administered might be thought to have a bearing on the uptake of the radiopharmaceutical. Table II shows the normalised counting rates obtained at 24 h post-injection, excluding patients in whom any extravasation was noted. A total body count index was obtained by normalising the counts in the standard views obtained 24 h post-injection to a 100 s count time, summing the results and dividing by the amount of activity administered. It can be seen that there was no significant effect attributable to the variation in protein dose, even at the 200 $\mu g$ level.

More than 95% of the activity remaining in the blood was associated with the plasma fraction from 1 h post-injection onwards. The blood clearance data can be fitted by a bi-

**Table II** Total body uptake at 24 h versus protein dose

| | *200 μg* | *1 mg* | *5 mg* | *20 mg* |
|---|---|---|---|---|
| | 8.89 | 4.44 | 6.66 | 7.11 |
| | 7.47 | 6.62 | 6.89 | 3.45 |
| | 4.10 | 10.29 | 7.77 | 6.32 |
| | 6.32 | 7.03 | 6.62 | 7.11 |
| | 9.62 | 8.26 | 7.91 | |
| Mean | 7.22 | 6.90 | 7.17 | 6.00 |
| s.d. | 1.96 | 2.20 | 0.62 | 1.74 |

**Table III** Absorbed radiation dose estimates ($\mu$Gy MBq$^{-1}$)

| | |
|---|---|
| Liver | 513 |
| Spleen | 1,431 |
| Bone | 151 |
| Testes | 595 |
| Kidney | 684 |
| Total body | 95 |

exponential function, 62% of the activity having a half-time in blood of 9.6 h and the remaining 38% a half-time of 46.8 h.

Dosimetry was calculated according to the schema of the MIRD Committee. Organ uptake was assumed to occur instantaneously with the biodistribution of activity taken to be that found at 24 h post-injection. Other than the blood, biological half-life was taken as the physical half-life of the radionuclide. This will result in an overestimation of the dose, but the errors involved in the calculations do not justify more accurate quantification: there were substantial inter-patient differences in organ uptake. The resulting mean dose estimates are given in Table III.

The radiation doses are generally higher than those extrapolated from animal data, particularly for the testes, due to the cross-reactivity discussed above. The figures are in general agreement with those given by Taylor *et al.* (1988) for a protein dose of 2.5 mg of their antibody.

### Discussion

These results are similar to previously published studies, suggesting that using similar monoclonal antibodies which recognised the high molecular weight antigen, approximately 80% of melanoma deposits can be detected. Siccardi *et al.* (1986) suggest that with small lesions of a diameter of 1 cm or less this detection rate may fall to only 59%. These authors also suggest that the lung is the most difficult area from which to obtain accurate images. This correlates with our own experience, and it may be that altered blood flow dynamics in and around pulmonary metastases result in poor perfusion of lung metastases and thus inadequate uptake. Cerny *et al.* (1987) suggest that particularly reliable images are obtained when studying metastatic deposits in bone, liver and lymph nodes, while lung, stomach and bowel are difficult areas for imaging. The latter two sites may give rise to problems with interpretation because of antibody excretion via the gastrointestinal tract. Kirkwood *et al.* (1987) comment that a very similar monoclonal antibody is cleared faster in males than in females or in one castrated male in their study, and also observe the apparently non-specific concentration of antibody in testicular tissue. This study also reports false positive results obtained in bone, heart, breast and thyroid.

It is possible that SPECT imaging of the thorax and abdomen routinely at 24 h post-injection might contribute to further specificity and sensitivity of the imaging technique. Greater use of SPECT would also aid in the more precise localisation of soft tissue lesions. In particular, this might improve the diagnostic capability for lesions in lungs and liver. It should, however, be borne in mind that the individual reporting the images in this study had virtually no clinical information, whereas the CT scans were reported with full clinical details available. The accuracy of the imaging reports was noticeably improved in the latter patients.

An important observation in this study was that at protein doses of 1 mg and 200 $\mu$g, no detectable antimurine response was observed from serum samples taken 28 days post-injection. This important finding requires confirmation, but is encouraging in clinical situations where repeated use of foreign protein might be required. For example, this would allow imaging studies to be followed by therapeutic administration without accelerated clearance of the murine antibody. Patients 4 and 22 had tumour uptakes in the range 0.03–0.06% per gram, which would permit cell kill if the antibody were labelled with a beta- or alpha-emitting radionuclide. Strategies would be required to reduce the non-target uptake of antibody, particularly that in liver and spleen. In view of the very poor response of metastatic melanoma to conventional chemotherapy, this approach to the management of advanced disease is clearly worthy of investigation and is in progress.

The authors are grateful to Xoma Corporation for the supply of the XMMME-001 antibody and for support for this study. Thanks are due also to Mrs M. Watson, Mrs A. McCall, Mrs M. Malcolm and Mrs E. Miller for their excellent technical assistance, to Mr G.J. Gillen for tomography and to Dr G.M. McCurragh for dosimetry. The assitance of Dr J. Shaw Dunn in providing anatomical data for use in the dosimetric calculations is much appreciated.

### References

BURAGGI, G.L., CALLEGARO, L., MARIANI, G. & 12 others (1985). Imaging with $^{131}$I-labeled monoclonal antibodies to a high-molecular-weight melanoma-associated antigen in patients with melanoma: efficacy of whole immunoglobulin and its F(ab′)$_2$ fragments. *Cancer Res.*, **45**, 3378.

CERNY, T., OWENS, S.E., MACKENZIE, S.A. & 4 others (1987). Immunoscintigraphy with $^{99m}$Tc labelled F(ab′)$_2$ fragments of an anti melanoma monoclonal antibody (225.28S) in patients with metastatic malignant melanoma. *Eur. J. Nucl. Med.*, **13**, 130.

KIRKWOOD, J.M., NEUMANN, R.D., ZOGHBI, S.S. & 6 others (1987). Scintigraphic detection of metastatic melanoma using indium 111/DTPA conjugated anti-gp240 antibody (ZME-018). *J. Clin. Oncol.*, **5**, 1247.

LARSON, S.M. (1987). Lymphoma, melanoma, colon cancer: diagnosis and treatment with radiolabeled monoclonal antibodies. *Radiology*, **165**, 297.

LOTZE, M.T., CARRASQUILLO, J.A., WEINSTEIN, J.N. & 9 others (1986). Monoclonal antibody imaging of human melanoma. *Ann. Surg.*, **204**, 223.

SICCARDI, A.G., BURAGGI, G.L., CALLEGARO, L. & 15 others (1986). Multicenter study of immunoscintigraphy with radiolabeled monoclonal antibodies in patients with melanoma. *Cancer Res.*, **46**, 4817.

TAYLOR, A., MILTON, W., EYRE, H., CHRISTIAN, P. & 5 others (1988). Radioimmunodetection of human melanoma with indium-111 labelled monoclonal antibody. *J. Nucl. Med.*, **29**, 329.